# Proteomic Analysis of Anti-Tumor Effects of 11-Dehydrosinulariolide on CAL-27 Cells

**DOI:** 10.3390/md9071254

**Published:** 2011-07-14

**Authors:** Chih-I Liu, Cheng-Chi Chen, Jiing-Chuan Chen, Jui-Hsin Su, Han Hsiang Huang, Jeff Yi-Fu Chen, Yu-Jen Wu

**Affiliations:** 1 Department of Nursing, Meiho Universtity, Pingtung 91202, Taiwan; E-Mail: x00002177@meiho.edu.tw; 2 Department of Beauty Science, Meiho University, Pingtung 91202, Taiwan; E-Mail: x00002073@meiho.edu.tw; 3 Department of Food Science and Nutrition, Meiho Universtity, Pingtung 91202, Taiwan; E-Mail: x00000017@meiho.edu.tw; 4 National Museum of Marine Biology and Aquarium, Checheng, Pingtung 94446, Taiwan; E-Mail: x2219@nmmba.gov.tw; 5 Department of Biological Science and Technology, Meiho Universtity, Pingtung 91202, Taiwan; E-Mail: hhuang.adsl@msa.hinet.net; 6 Department of Biotechnology, Kaohsiung Medical University, Kaohsiung 80761, Taiwan; E-Mail: yifuc@kmu.edu.tw

**Keywords:** 11-dehydrosinulariolide, CAL-27 cells, proteomic analysis

## Abstract

The anti-tumor effects of 11-dehydrosinulariolide, an active ingredient isolated from soft coral *Sinularia leptoclados*, on CAL-27 cells were investigated in this study. In the MTT assay for cell proliferation, increasing concentrations of 11-dehydrosinulariolide decreased CAL-27 cell viability. When a concentration of 1.5 μg/mL of 11-dehydrosinulariolide was applied, the CAL-27 cells viability was reduced to a level of 70% of the control sample. The wound healing function decreased as the concentration of 11-dehydrosinulariolide increased. The results in this study indicated that treatment with 11-dehydrosinulariolide for 6 h significantly induced both early and late apoptosis of CAL-27 cells, observed by flow cytometric measurement and microscopic fluorescent observation. A comparative proteomic analysis was conducted to investigate the effects of 11-dehydrosinulariolide on CAL-27 cells at the molecular level by comparison between the protein profiling (revealed on a 2-DE map) of CAL-27 cells treated with 11-dehydrosinulariolide and that of CAL-27 cells without the treatment. A total of 28 differential proteins (12 up-regulated and 16 down-regulated) in CAL-27 cells treated with 11-dehydrosinulariolide have been identified by LC-MS/MS analysis. Some of the differential proteins are associated with cell proliferation, apoptosis, protein synthesis, protein folding, and energy metabolism. The results of this study provided clues for the investigation of biochemical mechanisms of the anti-tumor effects of 11-dehydrosinulariolide on CAL-27 cells and could be valuable information for drug development and progression monitoring of oral squamous cell carcinoma (OSCC).

## 1. Introduction

Oral squamous cell carcinoma (OSCC), the most widespread malignant neoplasm of the oral cavity, is the sixth most prevalent malignancy worldwide and the fourth most common cancer among the Taiwanese male population [1]. OSCC can be treated by surgical operation, chemotherapy, radiotherapy, and combinations of the treatments; however, patients usually have poor prognosis, *i.e*., 50% survival rate in 5 years [2,3]. Studies indicated that the metabolite from soft coral such as steroids, terpenoids [4,5], and diterpenes have antimicrobial, antitumor, and HIV-inhibitory activities [6]. Acylspermidines from the Okinawan soft coral, *Sinularia* sp. expressed strong cytotoxicity against human tumor cell lines along with the induction of apoptotic phenomena [7]. Three norcembrane-based diterpenoid, 1-epi-leptocladolide A, leptocladolides A, and 7E-leptocladolide A have been isolated from soft coral *Sinularia leptoclados*. They have shown significant cytotoxic activity against KB and Hepa59T/VGH cancer lines [8]. Despite the numerous reports, the mechanisms of anti-cancer effects of the extracts from soft coral are still not well understood.

**Figure 1:**
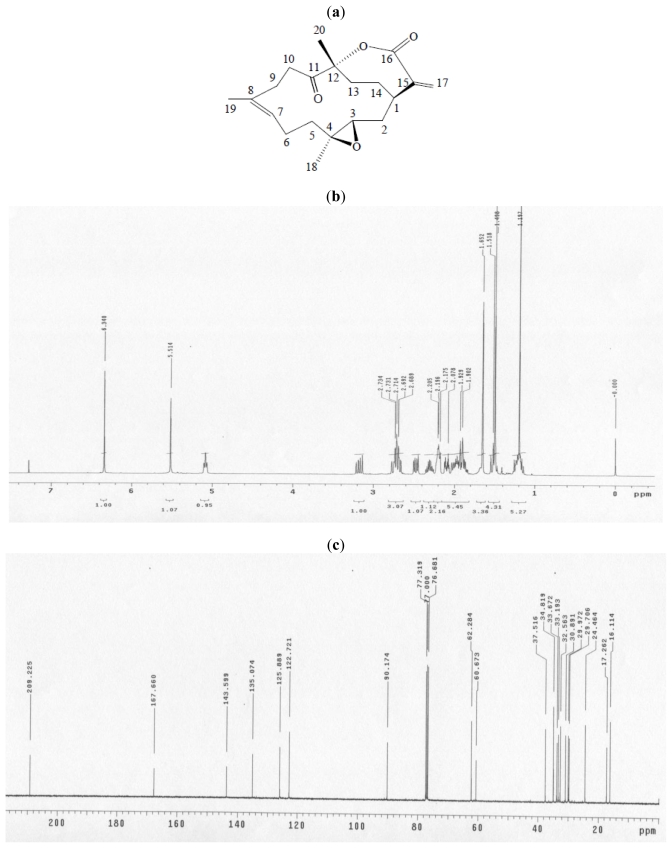
(**a**) Chemical structure of 11-dehydrosinulariolide; (**b**) 1H NMR spectrum of 11-dehydrosinulariolide in CDCl_3_ at 400 MHz; (**c**) 13C NMR spectrum of 11-dehydrosinulariolide in CDCl_3_ at 100 MHz.

Analysis of the protein profilings of the cells and tissues by proteomic analysis has been rapidly developed. Proteomic analysis provides a systematic approach to reveal the overall protein expression along with qualitative and quantitative analysis of a specific protein. Comparative proteomic analysis can be used to monitor the up-regulation and down-regulation of proteins under treatments with different compounds in comparison with the control. The results could provide valuable information for drug development and monitoring disease progression [9,10]. In this study, comparative proteomic analysis with two-dimensional gel electrophoresis (2-DE) was performed to investigate the effects of 11-dehydrosinulariolide on the oral caner cells. The chemical structure and the NMR spectra of 11-dehydrosinulariolide were shown in Figure 1. It is an active compound isolated from the soft coral *Sinularia leptoclados* [11]. Differential proteins were detected by comparing between the overall protein expression of CAL-27 cells treated with 11-dehydrosinulariolide and that of CAL-27 cells without treatment. The biochemical functions of the differential proteins were also explored.

## 2. Results and Discussion

### 2.1. The Cytotoxic Effects of 11-Dehydrosinulariolide on CAL-27 Cells

**Figure 2:**
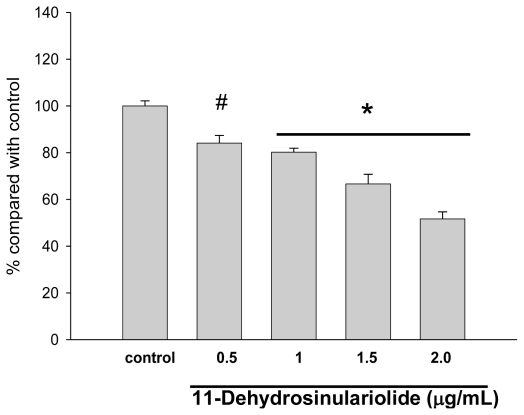
Viability of CAL-27 cells treated with 11-dehydrosinulariolide. Cells were treated with the indicated concentrations of 11-dehydrosinulariolide. Inhibition of cell proliferation was assessed by MTT assay as described in the Materials and Methods Section. The data were represented as the means ± SEM from three independent experiments (# *P* < 0.01 and * *P* < 0.001).

**Figure 3:**
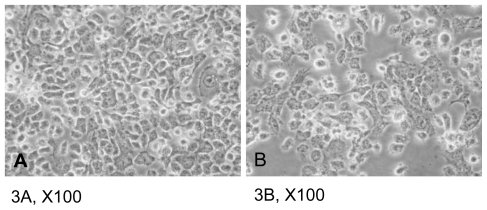
Morphological changes of CAL-27 cells treated with different concentrations of 11-dehydrosinulariolide for 24 h. (**A**) control sample (**B**) treated with 1.5 μg/mL of 11-dehydrosinulariolide.

**Figure 4:**
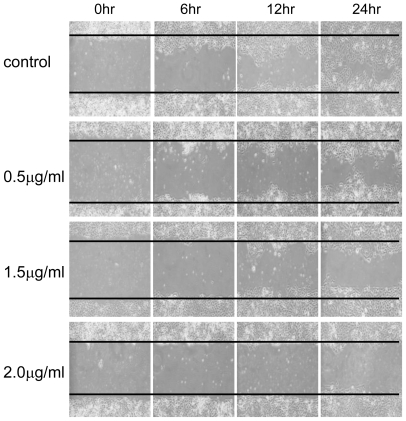
Wound healing assay of CAL-27 cells treated with 11-dehydrosinulariolide. The cancer cells in the areas between the two solid lines were those migrated in the gaps during the indicated time periods. Increased 11-dehydrosinulariolide concentration decreased the migration ability of CAL-27 cells.

The CAL-27 cells were treated with 11-dehydrosinulariolide of various concentrations (0.5, 1.0, 1.5, 2.0 μg/mL) for 24 h and the viabilities of the cells were determined by MTT assay. (Figure 2) The results indicated that increasing concentrations of 11-dehydrosinulariolide decreased CAL-27 cell viability. When a concentration of 1.5 μg/mL of 11-dehydrosinulariolide was applied, the CAL-27 cell viability was reduced to a level of 70% of the control sample. The morphological changes of the cells treated with 11-dehydrosinulariolide were observed by inverted light microscopy. The untreated cells that grew looked compact and smooth, on the other hand, the cells treated with 1.5 μg/mL of 11-dehydrosinulariolide showed shrinkage and a distinct decreased cell population compared with the counterpart (Figure 3). The effect of 11-dehydrosinulariolide on CAL-27 cell was also examined by wound healing assay. The wound healing function decreased as the concentrations of 11-dehydrosinulariolide increased (Figure 4).

### 2.2. Apoptosis of CAL-27 Treated with 11-Dehydrosinulariolide

**Figure 5:**
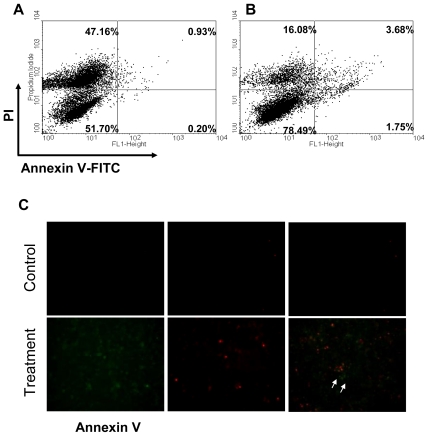
Flow cytometry analysis of the induced apoptosis of CAL-27cells treated with 11-dehydrosinulariolide by annexin V-FITC/PI expression. (**A**) CAL-27 cells were treated without 11-dehydrosinulariolide treatment; (**B**) The cells were treated with 11-dehydrosinulariolide at 1.5 μg/mL, then collected for staining with FITC-conjugated annexin V; (**C**) the fluorescently visualized cells were observed through florescent microscope.

To investigate the cytotoxicity effect of 11-dehydrosinulariolide for the induction of apoptosis, CAL-27 cells were treated with 11-dehydrosinulariolide and analyzed by flow cytometry. The apoptosis rate of 1.75% for CAL-27 was observed after the treatment with 1.5 μg/mL 11-dehydrosinulariolide for 6 h *versus* the apoptosis rate of 0.2% for the control sample (lower right of Figure 5). The late apoptosis/necrosis cell rate of 3.68% was observed for CAL-27 after the treatment with 1.5 μg/mL 11-dehydrosinulariolide *versus* the late apoptosis/necrosis cell rate of 0.93% for the control (upper right of Figure 5). These results clearly indicated that treatment with 11-dehydrosinulariolide significantly induced both early and late apoptosis of CAL-27 cells, determined by flow cytometric measurement and microscopic fluorescent observation (Figure 5C).

After treatment with 11-dehydrosinulariolide for 6 h, 1 × 10^6^ adherent cells were trypsinized and incubated with FITC-conjugated annexin V and propidium iodide(PI) for 15 min in the dark. Cells were analyzed by flow cytometry. In the gate areas, the upper right panel was for the population of late apoptotic cells and the lower right panel was for the early apoptotic cells with increased annexin V. The results indicated the treatment with 11-dehydrosinulariolide for 12 h significantly induced early and late apoptosis of CAL-27 cells.

### 2.3. Proteomic Analysis of CAL-27 Cells Treated with 11-Dehydrosinulariolide

**Figure 6:**
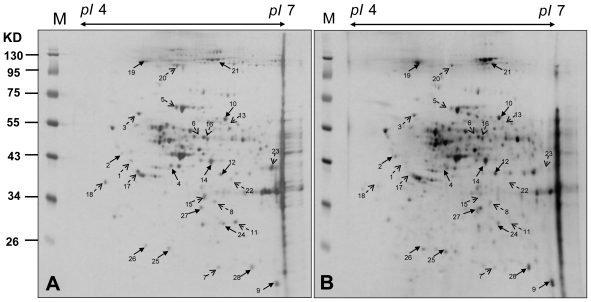
The 2-DE maps of CAL-27 cells treated with 11-dehydrosinulariolide and the control sample. (**A**) control (**B**) treated with 1.5 μg/mL 11-dehydrosinulariolide for 24 h. Proteins spots marked on the maps were considered differentially expressed and identified by LC-MS/MS. The results are representatives of three independent runs.

**Figure 7:**
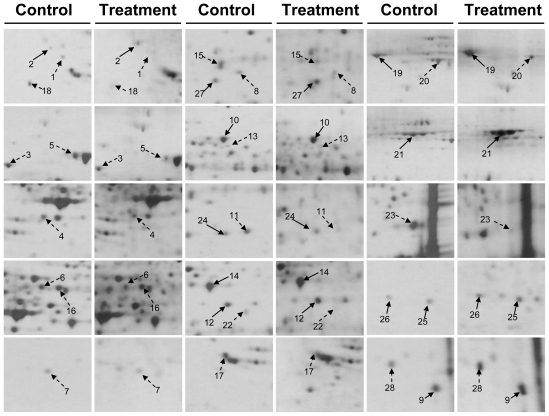
Enlarged 2-DE map of the 28 differential proteins in CAL-27 cells treated with 11-dehydrosinulariolide. Solid arrows point the up-regulated proteins and dashed arrows point the down-regulated proteins.

The CAL-27 cells were harvested after being treated with a 1.5 μg/mL solution of 11-dehydrosinulariolide. The proteins were extracted from cultured cells with lysis buffer. The supernatants were collected and the proteins were precipitated by TCA/Acetone. The 2-DE maps of the CAL-27 cells treated with 11-dehydrosinulariolide were compared with those of the control samples to examine the effect of 11-dehydrosinulariolide on CAL-27. The 2-DE were run with a loading of 50 μg protein (pI 4–7) and visualized by silver staining (Figures 6 and 7). PDQuest image analysis software (Bio-Rad) was employed for detecting the differential protein spots which were defined as the proteins showing a more than 1.5 fold intensity difference in 2-DE maps between the treated CAL-27 cells and the control samples. The protein identification was carried out by LC-MS/MS analysis after in gel digestion. MASCOT protein identification search software was used for the identification of the differential protein spots. A total of 28 differential protein spots were successfully identified. A list of the identified proteins with their MASCOT score, MS/MS matched sequences, apparent and theoretical MW, pI, coverage, and fold of change in expression level (up-regulation or down-regulation) were shown in Table 1.

The varied expression of proteins distributed throughout the entire gels indicated that multiple clusters of proteins were involved in the effects of 11-dehydrosinulariolide on CAL-27 cell. A total of 12 differential proteins were up-regulated after 11-dehydrosinulariolide treatment. They were protein SET, protein disulfide isomerase A3 (PDIA3), peptidyl-prolyl cis-trans isomerase A, UPF0160 protein MYG1, SPFH domain-containing protein 2 precursor, nucleolin, neutral alpha-glucosidase AB precursor, prohibitin, translationally-controlled tumor protein, inorganic pyrophosphatase, protein DJ-1, and lactoylglutathione lyase. A total of 16 differential proteins were down-regulated after 11-dehydrosinulariolide treatment. They were reticulocalbin-1, 78 kDa, glucose-regulated protein (GRP78), heterogeneous nuclear ribonucleoprotein F (hnRNP F), 60 kDa heat shock protein (Hsp60), actin-like protein 6A, proteasome subunit beta type 4 precursor, F-actin capping protein subunit beta, purine nucleoside phosphorylase, Xaa-Pro dipeptidase, 60S acidic ribosomal protein P0, ubiquinol-cytochrome-*c* reductase complex core protein 1 (UQCRC1), 40S ribosomal protein SA, transitional endoplasmic reticulum ATPase, isocitrate dehydrogenase subunit alpha, fructose-bisphosphate aldolase A, and nascent polypeptide-associated complex subunit alpha.

### 2.4. Validation by Western Blotting

**Figure 8:**
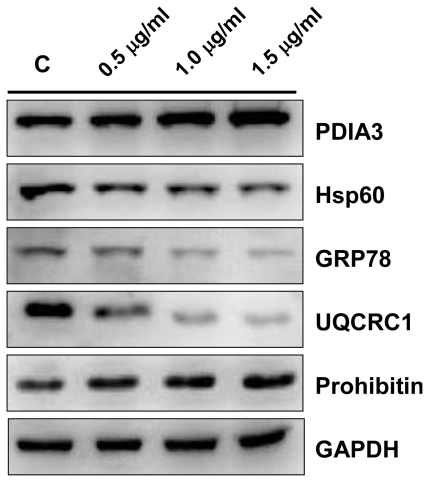
Western blotting analysis of PDIA3, Hsp60, UQCRC1, GRP78 and prohibitin of CAL-27 cells treated with increasing concentrations of 11-dehydrosinulariolide for 24 h. GAPDH was used as the internal control.

The identities of PDIA3, Hsp60, GRP78, UQCRC1 and prohibitin were further validated by western blotting (Figure 8). The results were in agreement with the 2-DE data.

### 2.5. Discussion

Comparative proteomic analysis can reveal the changes of protein expression of a proteome with a specific treatment by comparison with the control sample. The data could provide clues for the investigation of the effects of the specific treatment and the further understanding of the mechanisms at molecular level. There were reports about the antimicrobial, antiviral, and cytotoxic properties of soft corals [12–14]. It has also been reported that some chemical compounds from soft coral exert anti-tumor activities [8,15,16]. Most of the studies focused on the cytotoxicity of soft corals. There were fewer reports about the anti-tumor effects of active compounds in soft coral. In this study, the anti-cancer effects of 11-dehydrosinulariolide on CAL-27 cells by MTT assay, flow cytometry, and wound healing assay have been investigated. The proteins related with the effect of 11-dehydrosinulariolide have been identified by comparative proteomic analysis. The inhibitory effect of 11-dehydrosinulariolide on cell growth regarding dose-dependent, cytotoxicity or the induction of apoptosis were also assessed. A total of 28 differential proteins have been identified including 12 up-regulated proteins and 16 down-regulated proteins. It was found that some proteins such as PDIA3, Hsp60, GRP78, hnRNP F, UQCRC1 and prohibitin, are associated with apoptosis or inhibition of cancer cell growth.

#### 2.5.1. Protein Disulfide Isomerase A3 (PDIA3)

Protein disulfide isomerase A3, a member of the PDI family, participates in the oxidation, reduction, and isomerization of disulfide bonds for correct folding of secretory proteins before modification and transport in the endoplasmic reticulum [17]. It has been reported that PDI is induced by endoplasmic reticulum (ER) stress resulting from interference with Ca^2+^ homeostasis, inhibition of the formation of disulfide bond, and oxidative stress to relieve the accumulation of misfolded or unfolded proteins in the ER [18]. Study indicated that the accumulation of misfolded proteins in the ER initiates unfolded protein response (UPR) which adjusts ER protein folding capacity to promote cell survival. When homeostasis cannot be reestablished, apoptosis could be induced [19]. In the current study the up-regulation of PDIA3 (spot 10) was a response to ER stress resulting from 11-dehydrosinulariolide treatment. It is speculated the accumulation of misfolded protein caused apoptosis of CAL-27 cells.

#### 2.5.2. 60 kDa Heat Shock Protein (Hsp60)

Hsp60 has been shown to be connected with many aspects of cell functions such as protein folding and assembling of polypeptide chains in mitochondria [20]. Hsp60 exists widely in nature. It acts as chaperones to enhance cell survival under physiological stresses [21]. When Hsp60 is over-expressed, it protects cells from ischemia/hypoxic/oxidative injury to maintain mitochondrial integrity, to suppress mitochondrial membrane permeability, and to inhibit apoptotic and necrotic cell death [22]. It was reported that acute ablation of Hsp60 by small interfering RNA induces mitochondrial dysfunction, destabilizes mitochondrial pool of survivin, and activates both p53-dependent and mitochondrial apoptosis [23]. In this study, the down-regulation of Hsp60 after 11-dehydrosinulariolide treatment is an indication of the anti-cancer effect which may be related with p53-dependent or mitochondrial apoptosis in CAL-27 cells.

#### 2.5.3. 78 kDa Glucose-Regulated Protein (GRP78)

GRP78 (spot 3) was found to be down-regulation by 11-dehydrosinulariolide treated in the study. GRP78 is a constitutively expressed resident protein of the endoplasmic reticulum (ER) in all eukaryotic cells. It is a member of the highly conserved Hsp70 family and is involved in polypeptide translation, protein folding, and protein degradation [24]. Studies indicated that suppression of the stress-mediated induction of GRP78 could accelerate apoptosis, inhibit tumor growth, and increase the cytotoxicity of chronically hypoxic cells [25–27]. Down-regulation of GRP78 caused by interfering RNA leads to a slowdown of cell growth in glioma [28]. It has been reported that GRP78 could inhibit cytochrome *c*-mediated caspase activation in a cell-free system and caspase-mediated cell death [29]. It is speculated that the expression of GRP78 after the 11-dehydrosinulariolide treatment is related with caspase activation or ER stress response in CAL-27 cells.

#### 2.5.4. Heterogeneous Nuclear Ribonucleoprotein F (hnRNP F)

HnRNP F is highly expressed in tumor cells except in hepatocellular carcinoma [30]. It is a member of the hnRNP family which plays roles in transcription, RNA editing, and translation. HnRNP F is capable of catalyzing splicing events in both viral [31–34] and nonviral transcripts [35–37] and modulating the splicing of apoptotic regulator Bcl-x [35]. The elevated expression of Bcl-X_L_ is related to decreased apoptosis in cancer cells, increased risk of metastasis, increased resistance to chemotherapeutic drugs, and poor clinical outcome [38,39]. On the other hand, Bcl-X_s_ can induce apoptosis and help sensitize cells to chemotherapeutic agents [35].

In this study, the down-regulation of hnRNP F (spot 4) in CAL-27 cells after 11-dehydrosinulariolide treatment might be correlated with decreased expression of Bcl-X_L_ and increased apoptosis.

#### 2.5.5. Prohibitin

Prohibitins are evolutionary conserved proteins that are localized in mitochondria. They are also involved in transcriptional regulation [40,41], apoptosis [42,43], regulation of cellular signal [44], and mitochondrial biogenesis [45,46]. Translocation of prohibitin to mitochondria accompanies a simultaneous translocation of the p53 protein. It has been reported that p53 can translocate to the mitochondria in response to apoptosis [47–49]. It has been reported that prohititin has the potential to be an anti-proliferative protein, a tumor suppressor, or a regulator for cell progression and apoptosis [50]. The proteomic data in this study indicated that the expression of prohibitin was up-regulated in CAL-27 cells treated with 11-dehydrosinulariolide. This up-regulation is possibly associated with the induction of p53 export and the subsequent apoptosis.

#### 2.5.6. Ubiquinol Cytochrome *c* Reductase Complex Core Protein 1 (UQCRC1)

The expression of UQCRC1 (spot 16) was down-regulated in CAl-27 cells treated with 11-dehydrosinulariolide. UQCRC1, a key subunit of the cytochrome *bc*1 complex (complex III) of the mitochondrial respiratory chain, may mediate the formation of the complex between cytochromes *c* and *c*1 [51]. Complex III is responsible for generating an electrochemical potential coupled to ATP synthesis for transferring electrons from ubiquinol to cytochrome *c*. There were repots indicated that some mitochondrial myopathies were caused by deficiency of complex III [52,53]. Dysfunction of mitochondrial can result in excessive production of reactive oxygen species to stimulate the release of chtochrome *c* from mitochondrial intermembrane space to the cytosol and to trigger the transduction pathway of apoptosis [54,55]. It has been reported that mitochondrial oxygen radical formation could be regulated by the uniquinol/bcl redox couple [56]. The amplification or over-expression of UQCRC1 might create a stressful cellular environment. Energetic metabolism is another function of the mitochondrial respiratory chain. Therefore, amplification or over-expression of UQCRC1 might contribute to the rapid proliferation of cancer cells by rapid synthesis of ATP. The functions of some anticancer drugs are to inhibit electron transport in mitochondria at the levels of complex III [57,58]. Since UQCRC1 is a key subunit of complex III, modulation of this protein may contribute to cancer characteristics. Based on the results of this study, it is proposed that increased concentration of 11-dehydrosinulariolide resulted in a progressive inhibition of O_2_ uptake and decreased UQCRC1 expression in CAL-27 cells and, consequently, caused mitochondria dysfunction and subsequently triggered the transduction pathway of apoptosis.

## 3. Experimental Section

### 3.1. Cell Culture and Treatment with 11-Dehydrosinulariolide

CAL-27 cells were grown in DMEM with 4 mM L-glutamine adjusted to contain 1.5 g/L sodium bicarbonate and 4.5 g/L glucose, supplemented with 10% (v/v) FBS, 100 units/mL penicillin, 100 μg/mL streptomycin, 1 mM sodium pyruvate, and 0.01 mg/mL human transferring in a humidified atmosphere with 5% CO_2_ in air at 37 °C. When cells reached above 70% confluency, subculture was conducted at a split ratio of 1:6. CAL-27 cells were cultured in a 10 cm dish for each assay. The isolation of 11-dehydrosinulariolide from the soft coral *Sinularia leptoclado* was carried out according to the literature procedures [11]. Control cultures were made by adding DMSO at the same final concentration as in the treated samples (0.01% v/v). Cells were added with four different concentrations of 11-dehydrosinulariolide (0.5 μg/mL, 1.0 μg/mL, 1.5 μg/mL, and 2.0 μg/mL) and harvested after 24 h incubation. All the experiments were repeated three times to confirm reproducibility.

### 3.2. MTT Assay

The cytotoxic effect of 11-dehydrosinulariolide against CAL-27 cells was determined by the MTT assay. CAL-27 cells (1 × 10^5^/cm^2^) were incubated in 96 well microtiter plates. Treatment with various concentrations of 11-dehydrosinulariolide (0.5 μg/mL, 1.0 μg/mL, 1.5 μg/mL, and 2.0 μg/mL) followed for 24 h. Each well was added with 50 μL MTT solution (1 mg/mL in PBS) and the plates were incubated for 4 h at 37 °C. To achieve solubility of purple-blue MTT formazan crystals in viable cells, 200 μL of DMSO was aged to each well. The absorbance was read at 595 nm on a microtiter plate ELISA reader with DMSO as blank. Analysis of variance (ANOVA) followed by the Tukey-Kramer test on GraphPad InStat 3 (San Diego, CA, USA) to determine whether there were significant differences (*p* ≤ 0.05) [59].

### 3.3. Apoptosis and Cell Cycle Analysis

Apoptosis and cell cycle arrest induced by 11-dehydrosinulariolide for 6 h were determined by using an Annexin V-FITC and PI Apoptosis Detection Kit (BioVision, USA ) according to manufacturer’s instructions. Assessment of samples was accomplished FACSauto flow cytometer (Becton Dickinson, USA). Data analysis was carried out with WinMDI software (ver. 2.9, windows Multiple Document Interface, Flow Cytometry Application). The cells in the FITC-positive and PI-negative fractions were defined as apoptotic cells.

### 3.4. Wound Healing Assay

CAL-27 cells were plated in a 6-well plate. After the cells grew to confluence, a scratch/wound was made with a pipette tip in each of the wells. Cells were washed with PBS and refreshed with FBS-containing medium. Microscopic fields were photographed at the initial point (0 h) and thereafter.

### 3.5. Protein Extraction and Estimation

CAL-27 cells were untreated or treated with different concentrations of 11-dehydrosinulariolide (0, 0.5, 1.0 and 1.5 μg/mL) for 24 h and then lysed with Cell Extraction Buffer (BioSource International, Camarillo, CA, USA) and protease inhibitor cocktail (Sigma). The total protein in the supernatant was then precipitated out for overnight (−20 °C) by triple the volume of 10% TCA/Acetone solution containing 20 mM DTT. After centrifugation at 8000 rpm for 30 min at 4 °C, the supernatant was discarded. The pellet was suspended in a rehydration buffer (6 M urea, 2 M thiourea, 0.5% CHAPS, 0.5% IPG buffer, 20 mM DTT, and 0.002% bromophenol blue) at 4 °C for overnight. The IPG buffer was purchased from GE Healthcare. The protein contents were determined using 2-D Quant Kit (GE Healthcare).

### 3.6. Two-Dimensional Gel Electrophoresis

The first dimension electrophoresis (isoelectric focusing) was performed on GE Healthcare Ettan IPGphor 3 at 20 °C with a current limit of 30 A per strip. A sample was dissolved in a rehydration buffer as described above and applied on an IPG strip in a strip holder. Every 11-cm IPG strip (pI 4–7, Immobiline DryStrip) was rehydrated at 30 V for 12 h and then focused according to the preset program: 200 V (2 h), 500 V (2 h), 1000 V (2 h), 4000 V (1 h), 8000 V (4 h), until the total Vh reached 39,400. The equilibrated strip was placed on the top of a SDS-PAGE gel (12.5%), sealed with 0.5% agarose, then the second dimension electrophoresis was run at 150 V for 6.5 h. Electrophoretic unit used for the second dimension electrophoresis was SE 600 Ruby (Hoeffer). 2-DE images were made in triplicate for each sample and normalized prior to statistical analysis.

### 3.7. Protein Identification by LC-MS/MS

In-gel Digestion: A protein spot of interest was excised into a piece of 1 mm × 1 mm, then placed in a microcentrifuge tube. A 100 μL of 50 mM DTT in 25 mM ammonium bicarbonate (pH 8.5) was added to the tube which was shaken at 37 °C for 1 h. After removing excess DTT in supernatant, a 100 μL of 100 mM Iodoacetamide (IAA) in 25 mM ammonium bicarbonate (pH 8.5) was added to the tube which was then shaken for 30 min at RT in dark. The excess IAA in supernatant was removed. A 100 μL of 50% acetonitrile in 25 mM ammonium bicarbonate buffer (pH 8.5) was added and then the gel piece was soaked for 15 min followed a complete removal of the buffer. A 0.1 μg of trypsin in 10 μL 25 mM ammonium bicarbonate (pH 8.5) was added to the gel piece. Digestion was run for 16 h at 37 °C. The solution was sonicated for 20 s to release the tryptic peptides from the gel. The peptide solution was then concentrated for the LC-MS/MS analysis.

LC-MS/MS analysis: A peptide mixture was separated by nanoflow reversed phase C18 chromatography on nano LC using the Agilent 1200 System and PepMap100 C18, 75 μm × 15 cm (3 μm) nanoLC column or HPLC using the Agilent NanoLC 1200 System and Agilent Zobax 2.1 mm × 150 mm C18 column. LC-MS/MS analysis employed a 10 min online trapping and desalting step followed by a 60 min 5–40% mobile B gradient at nano flow and a 15 min 5–40% mobile B gradient at higher flow (mobile B = 98% ACN, 0.1% formic acid). Samples were analyzed on the AB SCIEX QTRAP^®^ 5500Q mass spectrometer (Applied Biosystems, CA, USA). The scan range was from *m/z* 100 to1000 for MS. The raw data was processed into a text file format of WIFF with Analyst 1.5.1.

### 3.8. Western Blotting Analysis

After SDS-PAGE analysis of the treated samples and the controls under reducing conditions, the proteins on gel were transferred to a PVDF membrane (Millipore) for 1.5 h at 400 mA using Transphor TE 62 (Hoeffer). The membranes were then incubated with rabbit polyclonal antibodies against human GRP-78, Hsp60, PDIA3, UQCRC1 and GAPDH (ProteinTech Group, USA) and rabbit monoclonal antibody anti human prohibitin(Epitomics, California, USA) at 4 °C for 2 h. The membranes were washed three times in PBST (10 mM NaH_2_PO_4_, 130 mM NaCl, 0.05% Tween 20), then probed with the second Ab (goat anti-rabbit and horseradish peroxidase conjugate, 1:5000 in blocking solution) for 1 h. After it was washed with PBST for three times, the enzyme activity on the blot was visualized through chemiluminesence by adding ECL Western Blotting Reagents (Pierce).

## 4. Conclusion

The anti-tumor effects of 11-dehydrosinulariolide on CAL-27 cells have been extensively investigated in this study. In the MTT assay for cell proliferation, the inhibition ratio increased as the concentration of 11-dehydrosinulariolide increased. When a concentration of 1.5 μg/mL of 11-dehydrosinulariolide was added to CAL-27 cells, the viability of cells was reduced to a level of 70% of the control. The effect of 11-dehydrosinulariolide on CAL-27 cell was also examined by wound healing assay. The wound healing function decreased as the concentration of 11-dehydrosinulariolide increased. The cytotoxicity effect of 11-dehydrosinulariolide for the induction of apoptosis of CAL-27 cells has also been studied. The results indicated that treatment with 11-dehydrosinulariolide significantly induced both early and late apoptosis of CAL-27 cells, observed by flow cytometric measurement and microscopic fluorescent observation. Comparative proteomic analysis provided an extensive and effective approach to detect changes of protein expression profiling of CAL-27 cells treated with 11-dehydrosinulariolide. A total of 28 differential proteins (12 up-regulated and 16 down-regulated) in the CAL-27 cells treated with 11-dehydrosinulariolide have been identified by LC-MS/MS analysis. The differential proteins, which were defined as the proteins showing a more than 1.5 fold intensity difference in the 2-DE maps between the treated CAL-27 cells and the control samples, including protein disulfide-isomerase A3, 78 kDa glucose-regulated protein, peptidyl-prolyl cis-trans isomerase A, prohibitin, heterogeneous nuclear ribonucleoprotein F, 60 kDa heat shock protein, ubiquinol cytochrome *c* reductase complex core protein 1, 40S ribosomal protein SA, transitional endoplasmic reticulum ATPase, fructose-bisphosphate aldolase A, and nascent polypeptide-associated complex subunit alpha. Some differential proteins are associated with cell proliferation, apopotosis, protein synthesis, protein folding, and energy metabolism.

The results of this study indicated that 11-dehydrosinulariolide could inhibit CAL-27 cell proliferation by exerting effects on regulating the expressions of some specific proteins. Identification and characterization of some functionally modulated proteins in anti-cell-proliferation could help understand the long-term effects of 11-dehydrosinulariolide on CAL-27 cells at the molecular level. Further studies of these proteins will help unveil the anti-tumor mechanisms of the treatment with 11-dehydrosinulariolide and thus benefit the development of new target drugs for oral squamous cell carcinoma (OSCC).

## Figures and Tables

**Table 1:** Identities of the differential proteins in CAL-27 cells treated with 11-dehydrosinulariolide analyzed by LC-MS/MS.

Spot no.	Protein name	Accessio n no.	Calculate Mr/pI	Peptide matched	Sequence covered %	MASCOT score	Regulation (fold-change) *
1	Reticulocalbin-1	Q15293	38.86/4.86	7	28	148	−2.7
2	Protein SET (Phosphatase 2A inhibitor I2PP2A)	Q01105	33.47/4.23	5	20	87	+2.2
3	78 kDa glucose-regulated protein precursor	P11021	72.28/5.07	8	8	152	−2.0
4	Heterogeneous nuclear ribonucleoprotein F	P52597	45.64/5.38	29	35	345	−2.1
5	60 kDa heat shock protein	P10809	61.02/5.70	43	46	753	−3.7
6	Actin-like protein 6A	O96019	47.43/5.39	19	23	83	−2.2
7	Proteasome subunit beta type 4 precursor	P28070	29.18/5.72	6	35	118	−2.1
8	F-actin capping protein subunit beta	P47756	31.33/5.36	22	31	242	−2.8
9	Peptidyl-prolyl cis-trans isomerase A	P62937	18.0/7.68	2	9	49	+2.3
10	Protein disulfide-isomerase A3 precursor	P14136	56.75/5.98	84	57	1168	+2.2
11	Purine nucleoside phosphorylase	P00491	32.09/6.45	9	23	208	−6.4
12	UPF0160 protein MYG1	Q9HB07	42.41/6.25	2	5	73	+3.8
13	Xaa-Pro dipeptidase	P12955	54.51/5.64	11	14	199	−2.0
14	SPFH domain-containing protein 2 precursor	O94905	37.81/5.47	17	6	104	+2.2
15	60S acidic ribosomal protein P0	P05388	34.25/5.71	88	44	933	−2.7
16	Ubiquinol cytochrome c reductase complex core protein 1	P31930	52.61/5.94	23	28	296	−2.6
17	40S ribosomal protein SA	P08865	32.83/4.79	72	51	930	−1.8
18	Nascent polypeptide-associated complex subunit alpha	Q13765	23.37/4.52	25	32	494	−3.1
19	Nucleolin	P19338	76.56/4.6	51	34	519	+4.1
20	Transitional endoplasmic reticulum ATPase	P55072	89.26/5.14	127	46	1117	−1.6
21	Neutral alpha-glucosidase AB precursor	Q14697	106.8/5.74	85	39	988	+3.0
22	Isocitrate dehydrogenase subunit alpha	P50213	39.56/6.47	35	28	413	−3.8
23	Fructose-bisphosphate aldolase A	P04075	39.39/8.0	103	60	1330	−9.0
24	Prohibitin	P35232	29.78/5.57	23	36	115	+2.4
25	Lactoylglutathione lyase	Q04760	20.7/5.24	52	56	344	+1.5
26	Translationally-controlled tumor protein (TCTP)	P13693	19.58/4.84	35	52	198	+2.0
27	Inorganic pyrophosphatase	Q15181	32.63/5.54	45	55	530	+3.0
28	Protein DJ-1	Q99497	19.87/6.33	38	65	238	+2.0

* Regulations (fold-changes) of differentially expression proteins are expressed at 24 h treatment of 11-dehydrosinulariolide.
